# Plant structure and function: Evolutionary origins and underlying mechanisms

**DOI:** 10.1093/plphys/kiac320

**Published:** 2022-07-01

**Authors:** Jill C Preston, Neelima R Sinha, Keiko U Torii, Elizabeth A Kellogg

**Affiliations:** Department of Plant Biology, College of Agriculture and Life Sciences, The University of Vermont, Vermont 05405, USA; Department of Plant Biology, College of Biological Sciences, University of California, Davis, California 95616, USA; Molecular Biosciences, College of Natural Sciences, The University of Texas at Austin, Texas 78712, USA; Donald Danforth Plant Science Center, St. Louis, Missouri, USA

Biologists still have much to learn about the proximate mechanisms underlying evolution of plant morphology, physiology, and development. This is particularly true for non-model taxa that show novel trait evolution, such as mechanisms to adapt to hot dry climates (Farrant and Moore, 2011; Pardo et al., 2020) or to consume nutritious insects (Thorogood et al., 2018). Insight into the hierarchy of mechanisms underlying various plant traits is already opening the door to new innovations, including breeding more sustainable and hardy crops (Crews and DeHaan, 2015; Scheben and Edwards, 2017; Lin et al., 2020), and has the power to help explain complex biological phenomena such as high evolvability, convergence, and extinction risk (e.g. Christin et al., 2012; Moray et al., 2015; Gray, 2018).

In this Focus Issue, we present eight *Update* articles that review our current understanding of the timing and mechanisms underlying the evolution of diverse plant structure and function. The first four focus on traits that evolved early in the history of land plants (embryophytes), and are hypothesized to have been critical for the vast radiation of plants that has made terrestrial life possible. The remaining four emphasize traits specific to the highly successful flowering plants (angiosperms). A number of *Research Articles* and *Research Reports* published in the recent past or future also accompany this Issue. These latter articles largely zoom in on specific plant groups, giving detailed examples of character variation caused by changes at several levels of organization: genes, proteins, gene expression, and regulatory circuits, hormones, and cell and tissue types.

## Ancient land plant innovations

A defining event in the Earth’s history was the colonization of land by multicellular plants ∼500 million years ago (Gerrienne et al., 2016; Morris et al., 2018). The subsequent diversification of land plants had profound effects on the Earth’s atmosphere, primary productivity, and carbon sequestration, and was to shape the emergence of many ecosystems on which humans and other organisms rely (Dahl et al., 2010; Kenrick et al., 2012; Lenton et al., 2012). Success of land plants has been attributed to a number of key structural and functional changes that have occurred over their evolutionary history, such as more efficient photosynthesis (Edwards, 2014), changes in developmental timing (Davies et al., 2013; Piao et al., 2019), and the origin of specialized roots, vasculature, meristems, seeds and flowers (Pires and Dolan, 2012; Harrison, 2017). Other, less well-recognized innovations include the ability of plants to interact with different microorganisms to exploit previously inaccessible niches (Rodriguez and Redman, 2008; Delaux and Schornack, 2021).

In this issue, Puginier et al. (2022) review the current state of knowledge on the role of mutualistic associations in the colonization of land by green plants. Embryophyte terrestrialization, aided by mutualistic symbiosis with arbuscular mycorrhizal fungi, improved nutrient and water uptake in the challenging newly colonized environment. Studies in both vascular and non-vascular embryophytes show that these symbioses are regulated by orthologous mechanisms, demonstrating that the most recent common ancestor of extant embryophytes engaged in a symbiotic association with Glomeromycota fungi. The authors propose intriguing parallels between lichenization and mycorrhizal symbioses. Outstanding questions include elucidation of improved drought tolerance mechanisms in plants associated with arbuscular mycorrhizal fungi, and the possible role of a core bacterial microbiome in green plant terrestrialization (Puginier et al., 2022).

Another functional acquisition attributed particularly to the success of vascular land plants (tracheophytes) is their ability to acquire and transport water in non-aqueous habitats. The presence of morphologically complex, lignin-fortified transporting tissues in the tracheophytes makes them the most highly represented group of plants on land, and the gain of vascular tissues has long been considered a sudden novel event. Woudenberg et al. (2022), however, propose an alternative hypothesis in their *Update* that transporting tissues evolved through gradual and stepwise modifications. The authors also suggest that conducting tissues have a deeper origin than previously thought, as inferred by their presence in several non-vascular plant lineages and a setophyte (liverworts and mosses) clade with conducting tissues that have elongated cells and exhibit a polar arrangement of water-conducting tissue outside and sugar-conducting tissue inside. A paleobotanical approach to examine fossil records provides additional implications. Recent molecular studies that have utilized/perturbed genes involved in hormone signaling and transport, as well as cell specification in vascular tissue in tracheophyte models, also provide evidence for the gradual acquisition hypothesis. However, since the model non-vascular plant *Marchantia polymorpha* lacks any form of differentiated conducting tissue, and *Physcomitrium patens* lacks food-conducting tissues (leptoids), more in-depth sampling and numerous models will be needed to explore this idea further (Woudenberg et al., 2022).

Continuing the theme of lignin deployment in land plant evolution, Emonet and Hay’s (2022)*Update* focuses on diverse uses that plants make of this complex polymer, whose structure and function vary both within and between plants (Emonet and Hay 2022). The paper highlights three major tissues where plants deploy lignin: the xylem, the Casparian strip, and the endocarp of fruits. In each case, lignin deposition is precise, cell-specific, and creates cells that have unique functions within the plant. In addition, the different roles of lignin characterize both ancient and recent cladogenetic events. Lignified xylem and the lignified Casparian strip characterize vascular plants (now known to be sister to the bryophytes, which lack well-developed vascular tissue). While lignification of the endocarp is common among angiosperms, Emonet and Hay (2022) describe a peculiar pattern of lignification in the endocarp of all species of *Cardamine*, which makes the fruits explosively dehiscent. The paper makes clear that the term lignification encompasses a variety of genetic, biochemical, and functional processes. Lignin is a collection of phenolics with slightly different physical properties and patterns of deposition; these aspects may be uncoupled in development and over evolutionary time to lead to unique structures and functions.

A conspicuous difference among groups of land plants is the degree to which the diploid sporophytic stage dominates the life cycle. Bryophytes (liverworts, hornworts, and mosses) tend to have a dominant haploid gametophytic stage, whereas the sporophytic stage is more conspicuous in most vascular plants, albeit to a lesser extent in ferns and allies (Sorojsrisom, 2022). Although aboveground growth in all land plants derives from indeterminate apical cells, it is interesting that apical growth occurs largely in the dominant life stage, and is initiated by a range of “meristems”, ranging from single cells to complex mounds of cells (Harrison and Morris, 2018). Historically, a lack of taxonomic resolution between the major land plant groups, as well as limited developmental studies in non-seed plants, has made it difficult to infer what the ancestral apical meristem looked like and the mechanisms underlying its diversification. Fouracre and Harrison (2022) capitalize on recent progress in these areas to suggest that the ancestor of land plants had a single-celled apical initial and that multicellular diploid meristems have evolved multiple times independently in vascular plants. They further explore available molecular data to assess whether multicellular diploid meristems evolved from moss-like sporophytic apical initials, bryophyte-like sporophytic intercalary meristems, or bryophyte-like gametophyte apical cells. Together, these data suggest a complex scenario whereby vascular plant meristems evolved through the integration of gametophyte networks into a partly conserved sporophytic program. However, the authors caution that inferences about ‘gametophytic’ function might be biased by lack of studies on bryophyte sporophyte development, and call for future work in this arena.

## Innovations among the angiosperms

The enormous radiation of angiosperms has been driven by many unique morphological and physiological adaptations. One such innovation is the evolution of carbon concentrating mechanisms (CCM). While much attention has been focused on the C_4_ pathway because it is the concentrating mechanism employed by crops such as maize (*Zea mays*) and sorghum (*Sorghum bicolor*) (Edwards 2014), another important CCM is Crassulacean Acid Metabolism, or CAM photosynthesis. The *Update* by Heyduk (2022) summarizes recent progress in understanding the physiological and genetic underpinnings of CAM. A central point of this paper is that both heat and drought increase photorespiration in the light, and that CAM photosynthesis is a process that mitigates both.

Heyduk (2022) draws valuable parallels between the warm environments of the Miocene when CAM originated and the current and predicted climate of the Anthropocene. Although the current rise in CO_2_ levels might be expected to reduce the advantage of CAM photosynthesis, the accompanying increase in temperature and decrease in precipitation are sources of stress. Because CAM is also a stress-response mechanism, its value in a future environment is hard to predict. CAM plants respond differently to increased CO_2_ depending on the details of their CAM physiology. Movement of malate, the carbon storage molecule of CAM, is also critical for the function of the pathway. Heyduk points out that the vacuole, the vacuolar membrane, proton pumps, and malate transporters are all involved in this aspect of the pathway, with high-temperature responses not fully understood. The effect of higher temperatures on enzymes is also discussed, with the valuable observation that temperature effects on transcription and translation also need to be evaluated. One remarkable aspect of CAM plants is their ability to withstand high temperatures and drought for months without degradation of their photosynthetic apparatus. Further understanding of this and other stress tolerance responses is likely to inform the use of CAM as a means for crop development for future warmer climates.

Plant responses to temperature are also at the center of the *Update* by Preston and Fjellheim (2022), in which the authors highlight the complex connection between temperature and flowering time. Precise control of flowering (and hence fruiting) time is essential for plant survival in the wild and utility in cultivation. Such temperature-regulated flowering traits have arisen independently in different groups of plants over evolutionary time. The paper summarizes recent work on temperature sensing in plants, noting that the mechanisms for sensing ambient, low and high temperatures involve distinct signaling pathways. The work has mainly been done in Arabidopsis (*Arabidopsis thaliana*, Brassicaceae), leaving open many possibilities for future work in other systems. The paper also highlights the considerable disconnect between work on Arabidopsis, which has focused heavily on FLOWERING LOCUS T (FT), PHYTOCHROME B (PHYB), and their regulators; and work on cereal grasses (Poaceae), which has focused instead on proteins containing CCT domains, such as PHOTOPERIOD1 and VERNALIZATION1, the latter a MADS-box transcription factor unrelated to the Arabidopsis protein of the same name. One obvious direction for future work is to explore the functions of the Arabidopsis proteins in cereals and vice versa, and to pursue critical experiments on their responses to temperature.

More important, as pointed out by Preston and Fjellheim (2022), is investigation in a wider range of species spanning broad ecological tolerances. While the interaction of temperature and other controls of flowering has been studied in exquisite detail in some species, others have been scarcely investigated at all. The level of variation even within the model systems Arabidopsis and *Brachypodium distachyon* (Poaceae) is high. While the central members of flowering networks are conserved, their regulatory interactions often are not. Identifying general patterns thus will require extensive work in a variety of wild and cultivated plants in both laboratory and field settings.

Transitioning from plant physiology to plant functional morphology, Freund et al. (2022) delve into the fascinating world of carnivorous plants. The authors describe the trapping and secretory structures seen in various lineages of carnivorous plants and raise three important questions. What are the cell types in non-carnivorous ancestors that gave rise to carnivorous glandular cells? What molecular evolutionary mechanisms led to the convergent co-option of protein families involved in gland functions? What was the fate of ancestral cellular functions when glands were repurposed for carnivory? Carnivorous plants survive in nutrient-poor environments by trapping small organisms, digesting them, and absorbing nutrients released. These nutrient acquisition capabilities have evolved convergently across multiple angiosperm lineages over tens of millions of years in epidermal glandular structures of modified plant organs (usually leaves). The multiple independent occurrences of plant carnivory suggest that a wide range of angiosperm lineages had the genetic ability to transition from the non-carnivorous to carnivorous state. Whereas some carnivorous plants digest their prey by trapping associated microorganisms, most traps have secretory glands that secrete ions for acidification of the trap fluids, as well as a slew of enzymes including proteases, chitinases, and nucleases that dissolve the prey. The most commonly secreted proteins in glands of carnivorous plants are pathogenesis-related proteins, and of the 19 non-carnivorous plants tested 15 species showed protease activity in their glandular secretions. Freund et al. (2022) suggest that glandular functions in trap leaves could be convergent exaptations of the structures and exudates found throughout the angiosperms.

As highlighted by the evolution of carnivorous plants, the origin of morphological novelty is a fundamental challenge for evolutionary biology. Much has been made of the role of evolution as tinkering with existing structures, but novel structures are harder to explain. In their *Update*, Wang et al. (2022) summarize recent data on the nature of the grass spikelet, a tiny cluster of highly modified flowers that forms the terminal unit of most grass inflorescences. They highlight the diagnostic features of the spikelet, in particular a pair of bracts (glumes) that lack meristems in their axils; distal to the glumes are one or more bracts (lemmas) that subtend flowers. The genetic control of spikelet identity is thus control of glume and lemma identity. Many of the proteins that control spikelet identity are transcription factors and members of the same gene families that control floral identity in eudicots. In particular, transcription factors in the MADS-box and AP2 families, which are deployed in many angiosperms to control floral organ identity, are also central to specify appropriate development of grass spikelets. A connection with cytokinin has also been demonstrated. Two of the most phylogenetically distinct members of the grass family, *Streptochaeta angustifolia* and *Pharus latifolius*, from subfamilies that are successive sisters to the rest of the family, now have complete genome sequences available; data from those genomes will help understand the evolutionary origin of the spikelet.

The collection of papers in this Focus Issue celebrates the inclusion of diverse study systems, methodologies, and approaches, which together enable our understanding at the mechanistic level for gaining deep insight into structural and functional traits upon which plants, and humans alike, rely. We hope that this compendium will inspire future comparative studies into the weird and wonderful life of plants, and highlight the role of these types of studies in addressing pressing societal issues, such as climate change and food insecurity.
